# Injuries, Death, and Disability Associated with 11 Years of Conflict in Baghdad, Iraq: A Randomized Household Cluster Survey

**DOI:** 10.1371/journal.pone.0131834

**Published:** 2015-08-07

**Authors:** Riyadh Lafta, Sahar Al-Shatari, Megan Cherewick, Lindsay Galway, Charles Mock, Amy Hagopian, Abraham Flaxman, Tim Takaro, Anna Greer, Adam Kushner, Gilbert Burnham

**Affiliations:** 1 Al Munstansiriya University, College of Medicine, Baghdad, Iraq; 2 Human Development and Training Center, Ministry of Health, Baghdad, Iraq; 3 The Johns Hopkins Bloomberg School of Public Health, Baltimore, Maryland, United States of America; 4 Faculty of Health Sciences, Simon Fraser University, Burnaby, BC, Canada; 5 Harborview Injury Prevention and Research Canter, University of Washington, Seattle, Washington, United States of America; 6 School of Public Health, University of Washington, Seattle, Washington, United States of America; 7 School of Medicine, University of Washington, Seattle, Washington, United States of America; Örebro University, SWEDEN

## Abstract

**Background:**

The objective of this study was to characterize injuries, deaths, and disabilities arising during 11 years of conflict in Baghdad.

**Methods:**

Using satellite imagery and administrative population estimated size for Baghdad, 30 clusters were selected, proportionate to population size estimates. Interviews were conducted during April and May 2014 in 900 households containing 5148 persons. Details about injuries and disabilities occurring from 2003 through May 2014 and resultant disabilities were recorded.

**Findings:**

There were 553 injuries reported by Baghdad residents, 225 of which were intentional, and 328 unintentional. For intentional injuries, the fatality rate was 39.1% and the disability rate 56.0%. Gunshots where the major cause of injury through 2006 when blasts/explosions became the most common cause and remained so through 2014. Among unintentional injuries, the fatality rate was 7.3% and the disability rate 77.1%. The major cause of unintentional injuries was falls (131) which have increased dramatically since 2008, followed by traffic related injuries (81), which have steadily increased. The proportion of injuries ending in disabilities remained fairly constant through the survey period.

**Interpretation:**

Intentional injuries added substantially to the burden of unintentional injuries for the population. For Baghdad, the phases of the Iraqi conflict are reflected in the patterns of injuries and consequent deaths reported. The scale of injuries during conflict is most certainly under-reported. Difficulties recalling injuries in a survey covering 11 years is a limitation, but it is likely that minor injuries were under-reported more than severe injuries. The in- and out-migration of Baghdad populations likely had effects on the events reported which we could not measure or estimate. Damage to the health infrastructure and the flight of health workers may have contributed to mortality and morbidity. Civilian injuries as well as mortality should be measured during conflicts, though not currently done.

## Introduction

Injuries are increasingly recognized as a major component of the global burden of diseasebut little is known about civilian injuries, deaths and disabilities during conflict. [1] Between 1990 and 2010 road traffic crashes, falls, burns, violence, homicide, suicide, and war resulted in more than 5.1 million deaths annually.[2] However, these fatal injuries comprise only a fraction of the total number of injured, many who are left with substantial disabilities.[3]

While community-based injury data exist for stable low and middle income countries (LMICs) situations, little is known about civilian injuries in conflict.[4–16] During conflict, civilians may be inadvertently or intentionally injured. Control and governance measures which could contain risks may have failed. Access to medical services and their quality are often reduced, and professional staff may have fled.[17,18] Better data are needed to identify prevention strategies and plan interventions.

A recent longstanding conflict is the situation in Iraq. The Iraq conflict has had many phases since the 2003 invasion, each with distinctive characteristics. The initial phases were characterized by extensive aerial bombardments, which was succeed by more ground combat, militia and criminal activity, and more recently by extensive vehicle-borne explosives. Population-based studies during these conflict years have focused on excess mortality estimates using scientific methods as well as media accounts.[19–22] During a relative lull in conflict, Donaldson *et al* in 2009, conducted a community injury survey using a 3-month recall period.[23] The study found an injury incidence of 54.9 per 1,000 person-years, a rate similar to neighboring countries. War-related injuries constituted 8.4% of injuries. One the intents of this study was to compare injuries and deaths over 11 year period of time in this protracted conflict with the three month assessment in 2009. We anticipated major differences in the nature of events recalled with the two approaches, but these differences have not been studied in conflict situations.

The goal of this study was to examine all forms of injuries, deaths, and disabilities and deaths by Baghdad residents from 2003 through May 2014. The resulting information could then be used by governments, non-governmental and international organizations to understand the effect of injuries on a population during conflict and to better prepare for future conflicts and help plan interventions.

## Methods

### Survey development and implementation

Building on experience from previous randomized two-stage cluster surveys conducted in Iraq, Rwanda, and Sierra Leone, a team of international and Iraqi members designed a survey focusing on injury, death and disability.[21,15,13] A questionnaire was developed based on the World Health Organization community injury survey guidelines, injury surveys in Ghana, and the Surgeons OverSeas Assessment of Surgical Need (SOSAS).[24,4,13,15] Once consensus was achieved, the final tool was translated into Arabic, back translated into English, and pilot tested in Baghdad.

An injury was defined as any intentional or unintentional physical harm that required medical care, whether received or not, and with or without an intervention, and which resulted in loss or reduction in normal activities for a period of time. A crush injury was defined as resulting from pressure or squeezing of a body part between objects, and a mechanical injury as resulting from a mechanical device or machine. A disability was defined as a limitation to normal activities from the injury described. Blasts/explosions were recorded as intentional if the informant felt deliberate harm was intended.

A household was defined as a group of persons living together in a dwelling with a separate outer door and a separate kitchen. Information about the household composition and injured household members were obtained from the head of the household or senior adult after verbal consent. Household members with disabilities who were capable of answering questions, and consented, were interviewed. The senior householder supplied information about injuries and disabilities of household members who were unable to answer questions or not present. Apartment houses are infrequent in Baghdad, and when they were present they are commonly limited to 2–4 units. When they were encountered the housing units were treated as independent households, in the same way as would be separate dwellings.

Two teams of four Iraqi interviewers plus a supervisor, received three days of training in April 2014. The eight interviewers (6 female, 4 male) and two supervisors were community medicine or family medicine doctors with previous experience in field data collection. The interviews were conducted between May 8 and June 1, 2014. The interviewers collected details about the geographic and anatomic locations of injuries, as well as mechanisms, and the intentionality to cause physical harm. Data on care obtained, surgery and inpatient care required, and any associated disabilities were also collected.

Households declining participation were noted and the next household substituted. For security reasons, all households in a cluster were completed in a single day. A household interview typically took 20 minutes, depending on events. A cluster required 7–9 hours to complete.

### Sample selection

A two-stage cluster sampling technique was used to randomly select a total of 30 clusters and a start household for each cluster. Clusters were assigned based on the population data for the 14 administrative units within Baghdad, using a probability proportional to estimated size (PPES) techniques. In each of the 30 clusters interviews were conducted in 30 individual households, beginning with a randomly-selected start household. This sample size was adequate to measure an injury rate of 1% with a 95% confidence level and a design effect of 2.

**Fig 1:**
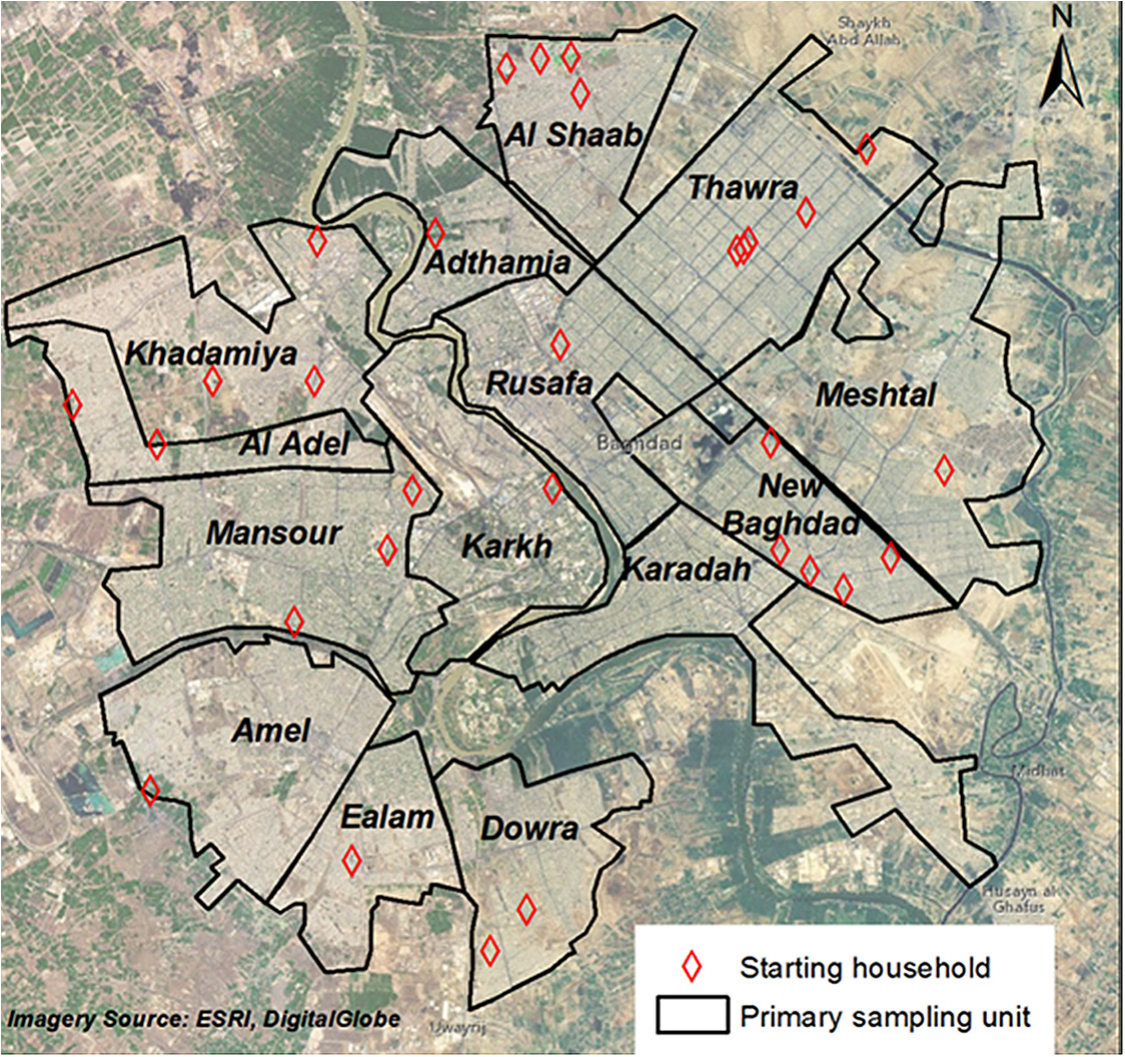
Cluster locations for the Baghdad injury survey.◊

Baghdad administrative unit population data were obtained from Iraq’s Central Organization of Statistics and Information Technology (COSIT), updated in 2011 (Table 1)[25] No attempt was made to adjust these figures for displacement or immigration since 2011. The second stage consisted of *a priori* and random selection of a cluster start household within the administrative units using satellite imagery, grids, and Google Earthmaps (Fig 1). Distribution of clusters is shown in Fig 1.

**Table 1:** Distribution of clusters.

Administrative unit	Estimated population	Number of clusters
Adthamia	280,319	1
Al Adel	563,019	2
Al Shaab	84,7387	4
Amel	311,143	1
Dowra	376,287	2
Ealam	286,712	1
Khadamiyah	633,797	3
Karadah	93,347	0
Karkh	226,694	1
Mansour	588,812	2
Meshtal	349,294	2
New Baghdad	1,068,270	5
Rusafa	171,484	1
Thawra	1,147,014	5
TOTAL	6,943,579	30

The sampling technique was adapted from the methods described by Galway *et al*. and Grais *et al*.[26,27] The cluster start households were physically located usually the day prior to interviews using detailed sampling maps. For each administrative area two backup clusters with start households were designated in the event that a primary cluster was in an unsafe neighborhood of if the start-household selected from satellite imagery was no longer a domestic residence.

Beginning with the start household, teams used a systematic sample technique including every other house until 30 households were interviewed. Baghdad streets are arranged in grids. When the residential street in the survey ended before 30 households were interviewed, interviewers turned right until completing 30 households, sampling only from residential streets.

### Statistical analysis

Paper forms were used and no names or addresses were collected. Data were entered in SPSS, and files were then individually rechecked against paper records and any discrepancies corrected. Descriptive statistical analysis was carried out on the full data set using Stata version 12 (College Station, TX). Subgroups were compared using chi squared and t-tests. Single and mixed effect logistic regression was used to estimate the influence of risk factors (age, sex, educational level, job category) on the outcome variable, injury (intentional and unintentional). The final multivariable model included sex and age (5 categories) on injury. Additional multivariable mixed effect models were used to estimate the odds of death and disability. These models included the independent variables age, sex, education, marital status and type of injury.

### Ethical approval

Ethical approval for the study was obtained from the Al Mustansiriya University scientific committee, and survey permission from the City of Baghdad. The Institutional Review Boards of Johns Hopkins Bloomberg School of Public Health and the University of Washington approved their faculty’s use of the anonymous database for secondary analysis. For security reasons only verbal consent was obtained, with the approval of the scientific committee, Verbal consent by participants was recorded by the interviewers. Interviews were conducted privately.

## Results

### Demography

From April–May 2014, 900 households visited, information was collected on 5,148 persons, both living and those dead from injuries. Three clusters were substituted, one for security reasons, and two because start houses were no longer residencies. In each case the first of the two pre-designated randomly selected back-up clusters was used. There were six households that declined to participate. Average household size was 5.72. Among all person in the study, 607 (12%) were under age 5, another 2,274 (44.2%) were aged 19 and younger, and 368 (7.2%) were 60 or over. Household composition was almost evenly divided between males and females (Table 2). These households comprised 45,442 person-years of exposure (Table 3).

**Table 2:** Household Composition and Injury by Age, Sex and Education, 2003–2014.*

**Household Size**	**N**	**(%)**				
1	14	1.6				
2	57	6.3				
3–4	246	27.3				
5–6	333	37.0				
7–8	130	14.5				
9–10	58	6.4				
11+	62	6.9				
**Mean (SD)**	5.7 (2.9)					
**Total**	900	100				
	**Males**		**Females**		**Total**	
**Age**	**N**	**(%)**	**N**	**(%)**	**N**	**(%)**
<5	301	11.7	306	11.9	607	11.8
5–19	859	33.3	808	31.5	1667	32.4
20–39	764	29.6	800	31.1	1564	30.4
40–59	483	18.7	459	17.9	942	18.3
60+	172	6.7	196	7.6	368	7.2
Mean (SD)	26.0 (19.2)		26.8 (19.7)		26.4 (19.4)	
Total	2579	50.1	2569	49.9	5148	100
**Among Injured**	**Intentional Injury**		**Unintentional Injury**		**Total Injuries**	
**Overall**	**N = 225**		**N = 328**		**N = 553**	
**Age**	**n**	**(%)**	**n**	**(%)**	**n**	**(%)**
<5	0	0.0	27	8.2	27	4.9
5–19	12	5.3	86	26.2	98	17.7
20–39	104	46.2	98	29.9	202	36.5
40–59	80	35.9	81	24.7	161	29.9
60+	29	12.9	36	11.0	65	11.8
Mean (SD)	40.2 (15.9)		31.5 (20.3)		35.0 (19.2)	
**Sex**						
Males	158	70.2	198	60.4	356	64.4
Females	67	29.8	130	39.6	197	35.6
**Education**						
None	21	9.3	81	24.7	102	18.4
Attended Primary	31	13.8	89	27.1	120	21.7
Completed Primary	70	31.1	71	21.7	141	25.5
Completed Secondary	56	24.9	44	13.4	100	18.1
Attended University	47	20.9	43	13.1	90	16.3
**Cause of Injury**	N	(%)	N	(%)	N	(%)
Blast or explosion-intention to harm harm	101	45.5	-	-	101	18.3
Gunshot	82	36.8	-	-	82	14.8
Other Intentional Trauma	32	14.2	-	-	29	5.2
Penetrating Wounds	5	2.2	-	-	5	0.9
Burns	4	1.8	-	-	4	0.7
Domestic Violence	3	1.3	-	-	3	0.5
Torture	1	0.4	**-**	**-**	1	0.2
Fall	-	-	131	39.9	131	23.6
Transport Related	-	-	81	24.7	81	14.6
Mechanical	-	-	53	16.2	53	9.5
Electrical Injury	-	-	14	4.3	14	2.5
Crush Injury	-	-	13	4.0	13	2.4
Poisoning	-	-	4	1.2	4	0.7
Blast or explosion (unintentional)	-	-	1	0.3	1	0.2
Eye Injury	-	-	1	0.3	1	1.2
Other Unintentional Injury	-	-	27	8.2	27	4.8
**Location of Injury**						
Open public space, street or square	161	71.6	109	33.2	270	48.8
House	20	8.9	166	50.5	186	33.6
Office or school	15	6.7	16	4.8	31	5.6
Industrial site, factory, shop	14	6.2	15	4.5	29	5.2
In a vehicle on a public road	7	3.1	15	4.5	22	4.0
Travelling by airplane or train	1	0.4	1	0.3	2	0.4
Farm	1	0.4	1	0.3	2	0.4
Other location	6	2.7	5	1.5	11	2.0
**Mortality**						
Death from Injury	88	39.1	24	7.3	112	20.3
**Disability**						
Disability from Injury	126	56.0	253	77.1	379	68.5

*Data from 2014 limited to first 3 to 5 months.

**Table 3:** Total injuries, injury rates per 1000 years of exposure and by type of injury, 2003–2014.*

		Intentional			Unintentional			Total Injuries		
* *		Injury Rate (per 1000 yrs)			Injury Rate (per 1000 yrs)			Injury Rate (per 1000 yrs)		
	Total person years exposed	Total Injuries	Rate	95 CI	Total Injuries	Rate	95 CI	Total Injuries	Rate	95 CI
***Overall***	45,442	225	5.0	4.3–5.6	328	7.2	6.4–8.0	553	12.2	11.2–13.2
***Sex***										
Male	22,782	158	6.9	5.9–8.1	198	8.7	7.5–10.0	356	15.6	14.1–17.3
Female	22,659	67	3.0	2.2–3.7	130	5.7	4.8–6.8	197	8.7	7.5–10.0
***Ages***										
<5	1,450	0	-	-	27	18.6	12.3–27.0	27	18.6	12.3–26.9
5–19	15,221	12	0.8	0.4–1.4	86	5.7	4.5–7.0	98	6.4	5.2–7.8
20–39	16,017	104	6.5	5.3–7.9	98	6.1	5.0–7.5	202	12.6	10.9–14.5
40–59	9,265	80	8.6	6.9–10.7	81	8.7	6.9–10.9	161	17.4	14.8–20.2
60+	3,490	29	8.3	5.6–11.9	36	10.3	7.2–14.3	65	18.6	14.4–23.7
***Education Level (Among Injured)***										
None	749	21	28.0	17.4–42.5	81	108.1	86.8–132.6	102	136.2	112.4–162.9
Attended Primary	1,147	31	25.3	17.0–36.1	89	79.3	64.4–96.5	120	104.6	87.5–123.8
CompletedPrimary	1,296	70	54.0	42.3–67.8	71	54.8	43.0–68.6	141	108.8	92.4–127.0
CompletedSecondary	871	56	64.3	48.9–82.7	44	50.5	36.9–67.2	100	114.8	94.4–137.9
Attended University	772	47	60.8	45.0–80.1	43	55.7	40.6–74.3	90	116.6	94.8–141.3

*Data from 2014 limited to first 3 to 5 months.

Men had twice the rate of intentional injury as women; however the nature of the injuries did not differ between sexes. Nearly three-quarters of intentional injuries occurred in public spaces. Half of unintentional injuries occurred in the home and a third in public spaces. Of intentional injuries, 88 or 39.1% ended in death and 56% resulted in disabilities. By contrast, only 24 (7.3%) of unintentional injuries were fatal but 77.1%% had some resulting disability.

From 2003–2014, there were 553 total reported injuries, 225 intentional and 328 unintentional (Table 3). The total injury rate was 12.2 (95% CI = 11.2, 13.2) injuries per 1000 person years of exposure. The highest reported injury rate was among those aged older than 60 years, 18.6 injuries per 1,000 years of exposure (95%CI 16.0–29.0). Men had a rate of 15.6 /1000 years (95%CI = 14.1, 17.3) and women 8·7/1000 years (95%CI = 7.5, 10.0). Among the population aged 60 and above there were 36 unintentional and 29 intentional injuries, For unintentional injuries, falls accounted for 23 (63.9%) and traffic injuries for 7 (19.4%). Gunshot wounds accounted for 15 (51%) intentional injuries and blasts or explosions 7 (24.1%).

Those with no education had the highest rates for unintentional injuries, 108.1/1000 years exposure (95% CI 86.8–132.6), or not having completed primary school, 79.3/1000 years (95% CI 64.4–96.5). Among intentional injuries, the highest rates were among those completing secondary school, 64.3/1000 years (95%CI 48.9–82.7).

### Intentional injuries

Among the 225 intentional injuries, the rate was 6.9/1000 person years among men, and 3.0 among women, with an overall rate of 5.0/1000 years. The highest rate for males was 13.9/1000 years among men aged 60–69, excluding the small number older than age 80. For women, the highest rates was 9.6/1000 years among those aged 50–59. Most injuries were caused by explosions (101) followed by gunshots (82), see Table 2. Explosions were a more common source of injury among males 78 (50.1%) than females 23 (35.4%). The majority of intentional injuries, 161 (71.6%), occurred in streets or open places, with smaller numbers in houses, offices or workplaces (Table 2). Of all 225 intentional injures, gunshot injuries affected 82 persons (Table 2): 59 males (37.8%) and 23 females (35.4%).

Injuries by type and by year are in Table 4. Gunshot injuries were most common from 2005–2007, but dropped rapidly later. Injuries from explosions were most common from 2006 to 2008, and again in 2013 and 2014. There were 14 injuries from explosions in the first five months of 2014, compared with 10 for all of 2013. Gunshots and explosions were the most common source of injuries in the 20–39 age group for men and the 40–59 age group for women, demographic details not shown.

**Table 4:** Intentional and unintentional injuries by year and type, 2003–2014.*

	Intentional Injuries n (%)				Unintentional injuries n (%)					
Injury Year	Gunshot Wound	Blast or Explosion	Other	Total	Fall	Crush	Mechanical	Traffic	Others	Total
2003	7 (8.5)	5 (5.0)	1 (2.4)	13 (5.8)	4 (3.1)	0 (0.0)	3 (5.7)	5 (6.2)	4 (7.5)	16 (4.8)
2004	4 (4.9)	4 (4.0)	5 (11.9)	13 (5.8)	6 (4.5)	1 (7.7)	2 (3.7)	4 (4.9)	1 (1.9)	14 (4.2)
2005	13 (15.9)	7 (6.9)	3 (7.1)	23 (10.2)	4 (3.0)	1 (7.7)	2 (3.7)	8 (9.9)	2 (3.7)	17 (5.1)
2006	20 (24.4)	10 (9.9)	9 (21.4)	39 (17.3)	2 (1.5)	0 (0.0)	1 (1.9)	5 (6.2)	2 (3.7)	10 (3.0)
2007	21 (25.6)	17 (16.8)	6 (14.3)	44 (19.6)	5 (3.8)	0 (0.0)	2 (3.7)	4 (4.9)	2 (3.7)	13 (3.9)
2008	6 (7.3)	13 (12.9)	2 (4.8)	21 (9.3)	2 (1.5)	0 (0.0)	3 (5.7)	4 (4.9)	4 (9.4)	14 (4.2)
2009	1 (1.2)	7 (6.9)	4 (9.5)	12 (5.3)	12 (9.2)	0 (0.0)	4 (7.6)	5 (6.2)	3 (5.7)	24 (7.3)
2010	3 (3.7)	5 (5.0)	4 (9.5)	12 (5.3)	14 (10.7)	2 (15.4)	5 (9.4)	6 (7.4)	8 (17.0)	36 10.9
2011	2 (2.4)	3 (3.0)	1 (2.4)	6 (2.7)	12 (9.1)	1 (7.7)	3 (5.7)	9 (11.1)	3 (5.7)	28 (8.5)
2012	3 (3.7)	6 (5.9)	4 (9.5)	13 (5.8)	20 (15.3)	1 (7.7)	13 (24.5)	9 (11.1)	6 (11.3)	49 (14.8)
2013	1 (1.2)	10 (9.9)	2 (4.8)	13 (5.8)	24 (18.3)	4 (30.8)	8 (15.1)	18 (22.2)	4 (9.4)	59 (17.8)
2014*	1 (1.2)	14 (13.9)	1 (2.4)	16 (7.1)	26 (19.9)	3 (23.1)	7 (13.2)	4 (4.9)	11 (20.8)	51 (15.4)
**Total**	82 (100)	101 (100)	42(100)	225 (100)	131 (100)	13 (100)	53 (100)	81 (100)	50 (100)	328 (100)

*Data from 2014 limited to first 3 to 5 months.

**Fig 2:**
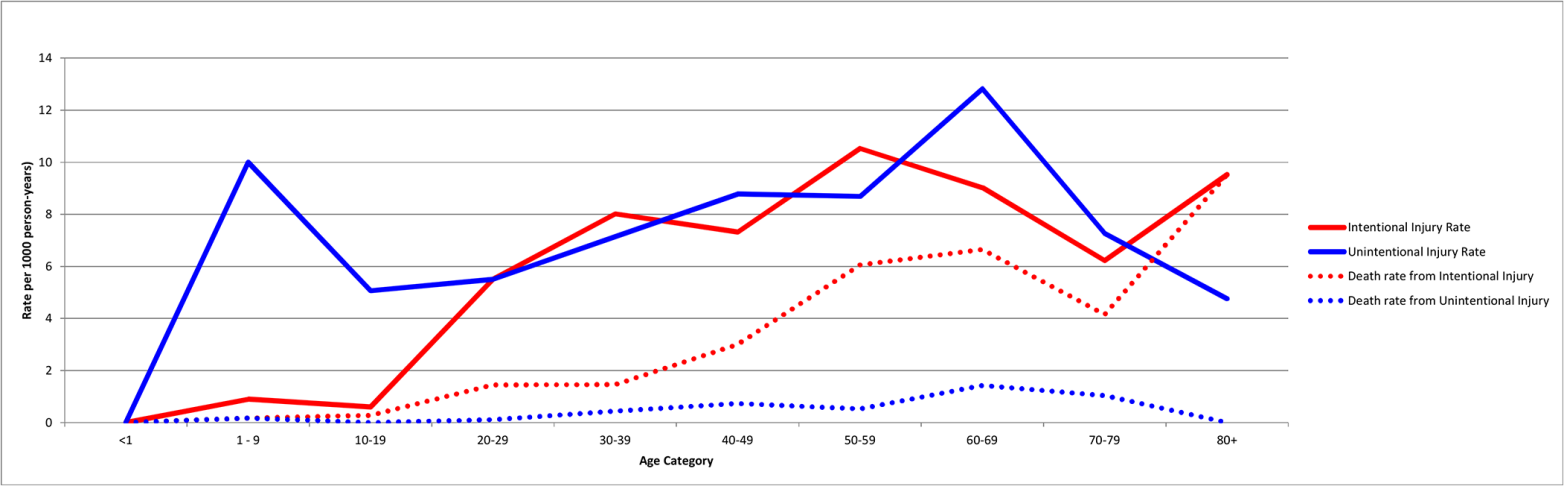
Injury, Disability and Death Rates by Age 2003–2014. Data from 2014 limited to first 3 to 5 months.

**Fig 3:**
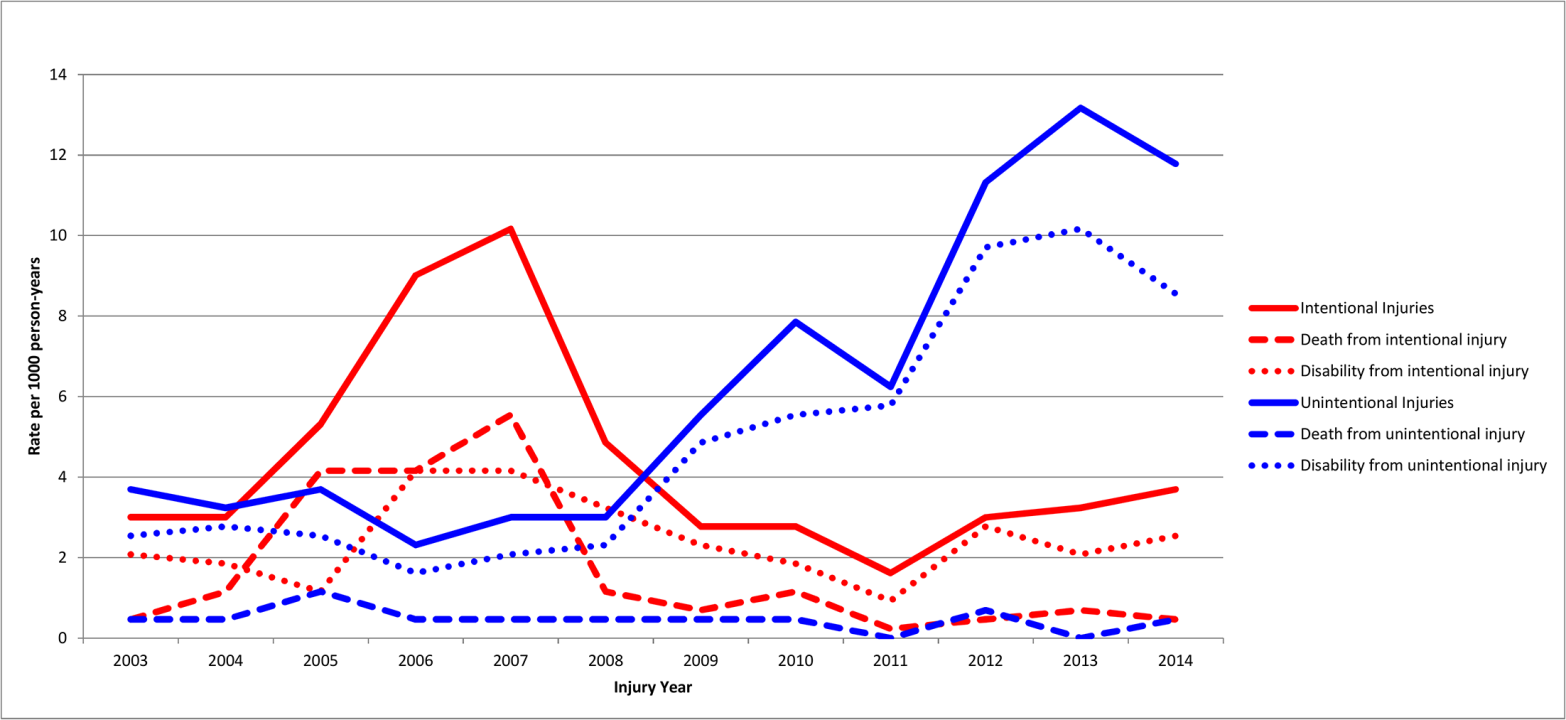
Injury Rates by Year and Outcomes, 2003–2014. Data from 2014 limited to first 3 to 5 months.

The majority of deaths from intentional injuries, 47 (55.3%), were among those with gunshot wounds, but the majority of disabilities, 74 (59.5%) were from blasts or explosions (Table 5). Rates for disabilities and deaths by age are in Fig 2 and by year in Fig 3.

**Table 5:** Injuries, deaths and disabilities, 2003–2014.*

	TotalN (%)	DisabilityN (%)	Death from InjuryN (%)	Death from other thanInjury N (%)**
**Intentional Injuries**				
Blast or Explosion	101 (44.9)	74 (59.5)	22 (22.3)	2 (100.0)
Gunshot	82 (36.4)	30 (24.2)	47 (55.3)	0 (0.0)
Other	42 (18.6)	22 (17.7)	19 (22.4)	0 (0.0)
**Total intentional Injuries**	225 (100)	126 (100)	88 (100)	2 (100.0)
**Unintentional Injuries**				
Fall	131 (39.9)	108 (42.7)	9 (37.5)	5 (18.3)
Transport related	81 (24.7)	64 (25.3)	6 (25.0)	0 (0.0)
Mechanical	53 (16.2)	45 (17.8)	1 (4.1)	0 (0.0)
Crush	13 (4.0)	8 (3.2)	0 (0.0)	0 (0.0)
Other	50 (15.2)	28 (11.1)	8 (33.3)	1 (16.7)
**Total unintentional Injuries**	328 (100)	253 (100)	24 (100)	6 (100)

*Data from 2014 limited to first 3 to 5 months.

** Cause of death unrelated to injury.

**Fig 4:**
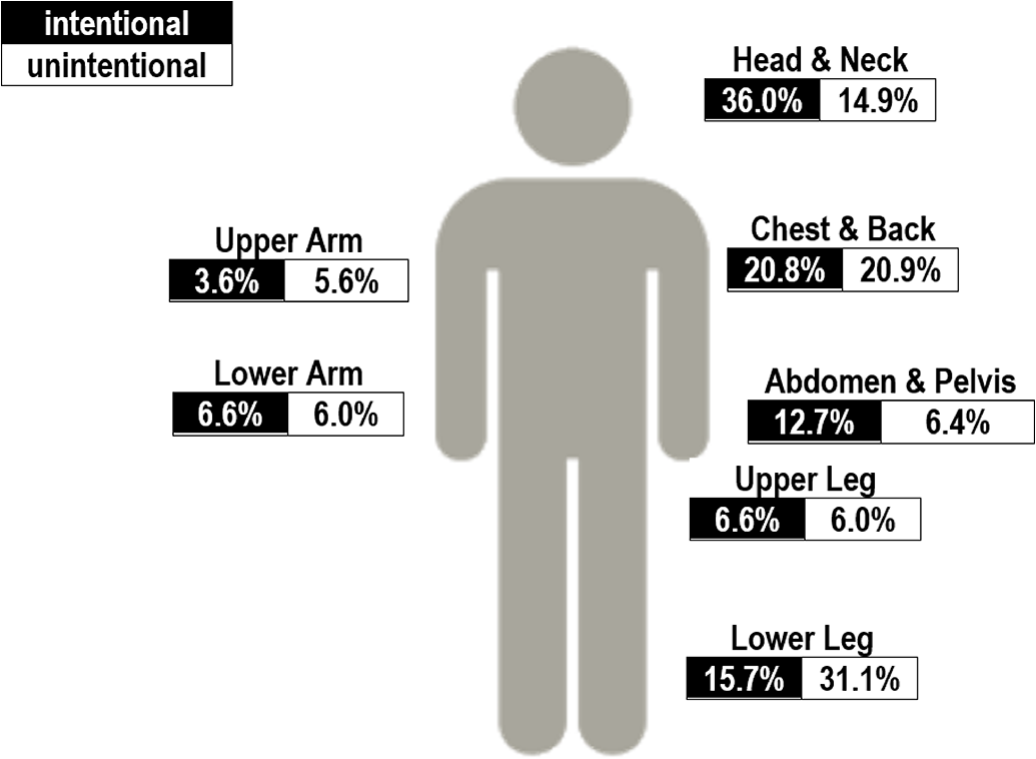
Anatomical Site of Injury by Type, 2003–2014. Data from 2014 limited to first 3 to 5 months.

The anatomical body region for intentional injuries was the head 119, (36.0%); the chest or back and/or spine, 69 (20.8%); lower leg, 52 (15.7%), and the abdomen and pelvis, 42 (12.7%), shown in Fig 4.

The odds of intentional injuries leading to deaths were highest during 2005–2007, a significant difference compared with other years (p<0.001). The odds ratio of risk of death from a blast injury was significantly less than gunshots (0.20, 95%CI = 0.11, 0.39, *p*<0.001) for all time periods combined. In Fig 2 death and disability due to intentional and unintentional injuries are depicted by year, with a surge in deaths 2005–2008.

### Unintentional injuries

Of unintentional injuries, 166 (50.5%) occurred in the home, with 109 (33.2%) occurring in streets or public spaces. Among the 328 unintentional injuries there were 24 deaths (7.3%) and 253 (77.1%) disabilities (Table 5).

The largest number of unintentional injuries, 131 (39.9%), were from falls. Other common causes were 53 mechanical injuries (16·2%), 13 crush injuries (4·0%), and 81 (24.7%) injuries which were transport related.

Mechanical injuries occurred mostly at home, but 68% of crush injuries occurred in streets or public places. Mechanical injuries preferentially affected the spine or back while crush injuries mostly affected the lower extremities. Transport related injuries mostly affected older children and young adults, and were evenly distributed across the years, with a small spike in 2012. Of transport related injuries 39 (48.2%) were as pedestrians, and 26 (27.2%) in automobiles. Injury rates per 1000 person years were consistently higher in males from childhood until age 50, at which the rates for females exceeded those of males (Table 2). There were 253 (77.1%) disabilities reported associated with unintentional injury.

**Fig 5:**
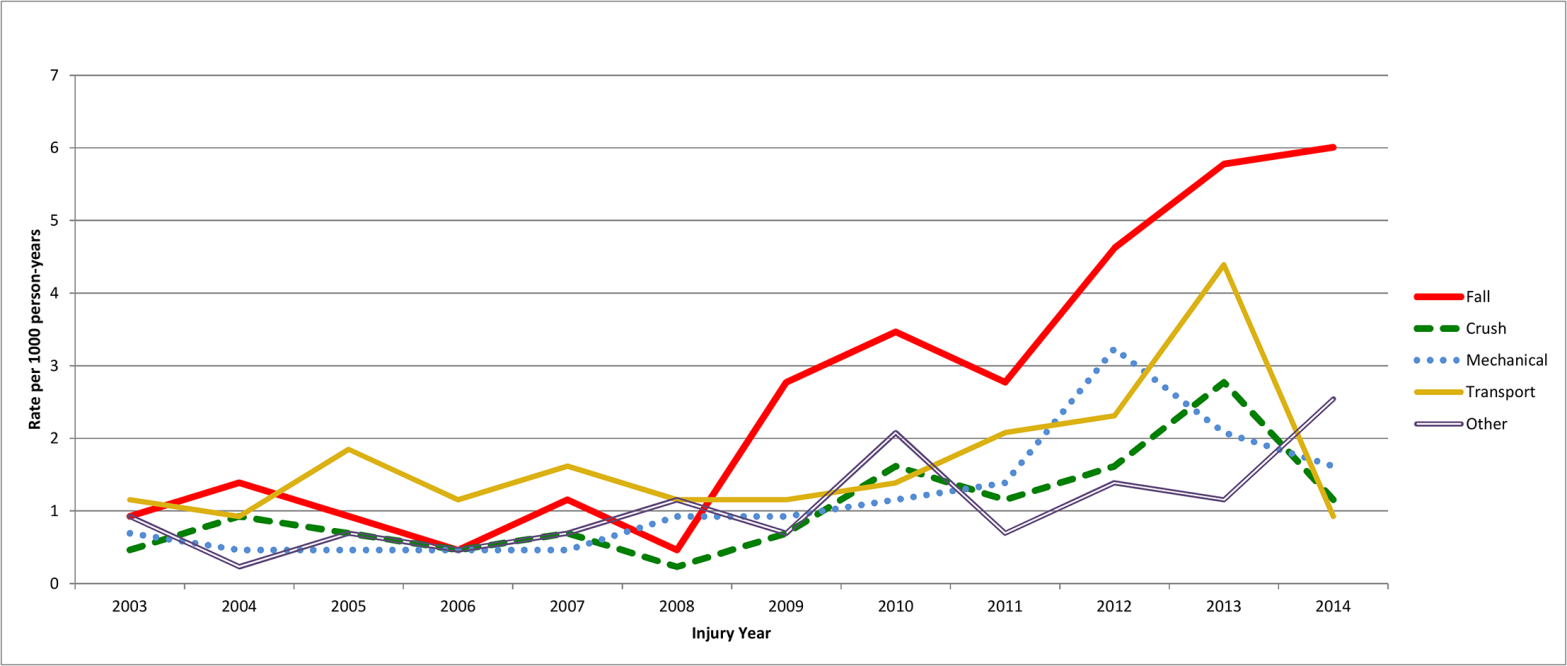
Unintentional injuries by type in Baghdad, 2003–2014. Data from 2014 limited to first 3 to 5 months.

The rate for reported unintentional injuries rose steadily from 2008, excepting a slight pause in 2011 (Table 4, Fig 2). While all unintentional injuries have tended to increase, the major driving force has been increasing numbers of falls (Fig 5).

### Injury care seeking behavior

The majority of persons sought hospital treatment after injury (N = 481): 52.5% of those with unintentional injuries and 52.8% with intentional injuries. Others sought care at public sector clinics and some at private clinics. Less than 5% did not seek formal medical care. Of those with intentional injuries 47.4% required surgery with a general anesthetic while for unintentional injuries this was 19.9%. Ten persons required amputations, five for intentional injuries and five for unintentional injuries.

### Falls

Falls were reported for 67 males (33.8%) and 64 females (49.2%). Two-thirds of falls were in a house, most of these among women. By contrast, men were commonly injured in falls occurring in public places, offices, school or industrial locations. Falls were common in all ages, but were most common among males under age 20. Of the 131 falls reported, 108 (82.4) resulted in some disability. Only nine falls ended fatally, but this was a quarter of all fatal unintentional injuries. Falls were less frequent before 2009, after which they increased each year except 2011. The increased falls occurred largely in houses, although there was some increase in falls in public places in 2012 and 2013. The most common body locations injured were the lower and upper extremities and the back or spine.

### Traffic related injuries

There were 81 various transport related injuries, accounting for 24.7% of all unintentional injuries. Of these, 37 occurred in motor vehicles, including bus (8), taxi (8), private car (14) and cycles (7). There were 39 pedestrian injuries. Transport related injuries were variously crush, burns and mechanical injuries. The most common types of injuries were fractures. The numbers of transportation injuries rose to 2 per 1000 years of exposure in 2005 and then decreased until reaching this number again 2011. The 2011 rate doubled for 2013, driven in part by an increase in pedestrian injuries, as well as some increase of injuries in automobiles and taxis, see Table 4.

## Discussion

This study describes the effect of conflict on injury patterns in Baghdad from 2003. While unintentional injury rates have held stable for most of the period, intentional injures have fluctuated in parallel with the stages of the conflict. Of the 553 total injuries reported, 225 or 40.7% were intentional injuries, which constituted 78.5% of all injury deaths, and 33.2% of all disabilities arising from injuries. These injuries are a burden on a damaged health service.[17, 18, 28, 29]

The pattern of deaths from intentional injuries follows the pattern of the Iraq conflict.[30] After the intense conflict of 2004–2008, intentional injuries decreased and their nature changed from gunshots to blasts and explosions. This is consistent with the findings of the population-based Iraq mortality studies.

The study findings showed that 39% of intentional injuries resulted in death while only 7.3% of unintentional injuries were fatal. Virtually everyone surviving the initial injury received medical care. The finding that half of intentional and a fifth of unintentional injuries required surgery with a general anesthetic suggests that many injuries reported were of a serious nature. Deterioration infrastructure, health worker loss, and poor quality of care may have increased mortality and morbidity after injuries.

Educational levels seemed to divide the injured populations with illiterate and less educated persons having a higher rate of unintentional injuries and more educated persons having higher rates of intentional injuries. Higher rates of intentional injuries among educated persons may reflect the specific targeting of educated and professional persons by militias or criminals.[18]

Unintentional injury rates remained relatively stable until 2009, when they began rising dramatically. Disabilities rates tracked injury rates closely and consistently throughout the study period, suggesting that injuries reported were not trivial. It could be that some of the rise in injuries from 2013/2014 is more apparent than real because of differential recall.

Persons with unintentional injuries were nearly 8.5 years younger than those with intentional injuries, with larger numbers in the under 20 age group. Women were more likely to be injured by falls, and poisonings were slightly more common in women. All other categories of unintentional injuries were more common in men.

During times of conflict and tensions, families tend to stay home and indoors which may explain levels remaining low until 2008. Transport injuries might be expected to rise as intense conflict ebbed, and people moved more freely, abetted by the rare use of automobile seatbelts and little concern for drivers’ licenses. However, the many police check points, blocked roads, and congested traffic, restricted speeding. Increased pedestrian injuries could come from more people on the streets as the security improved somewhat after 2008/2009. There were reports that coalition military vehicles travelling at speed often collided with passenger cars, possibly increasing transport related injuries in 2004–05.

The driver for the general rise in unintentional injuries is an increase in falls, even allowing for better recall of recent events. This trend was also noted by Donaldson in Baghdad in late 2009.[23] Falls are a frequent cause of trauma in many low and middle income countries, but some circumstances in Baghdad could differ. There has been rapid growth of temporary housing with an influx of families into Baghdad, especially in Sadr City. Families from elsewhere in Baghdad live here while rebuilding damaged primary residences. Many of these dwellings are unsafe, and falls common. This is especially from the flat un-railed roofs of houses which are often used as play areas for children and for cooking. In the hot evenings families gather here when there is no electricity for air conditioning. Inadequate lighting from power cuts may contribute to interior falls. Falls may not necessarily be from a height, but from tripping or stumbling. Finally, “falling down the stairs” is used as a cover for domestic violence injuries when presenting to hospital. Undoubtedly alcohol and prescription drug abuse play a role in the injuries reported, but this role could not be measured in this type of survey.[31]

Injury patterns would have been different if rural agricultural populations were part of the study which was limited only to urban Baghdad. Surprisingly missing from the list of unintentional injuries are burns, although these may have been included in blasts, explosions or traffic injuries.

Compared with injury reports from other countries in the region, the rates for non-intentional injuries we recorded are low.[10,32–34] In most cases the recall periods for the regional studies ranged from 3–12 months, rather than the 11 years in this study, and the inclusion criteria varied from self-reported to indications for surgical treatment. Injury definitions varied considerably among reports.

### Limitations

This type of study has many limitations. While the sampling process was random, it might not be completely representative. The sample did however, closely match the age and sex structure estimates for Iraq.[25]

Without comprehensive hospital trauma registries or health systems records spanning this time, surveys of randomly selected households are an alternative measure of the injury burden. The 11 year span of the conflict is long and many of the less serious injuries and short term disabilities will have been forgotten. The injuries and disabilities reported in this study are likely to be the more severe, particularly those directly or indirectly related to conflict, as more memorable. Mock *et al* showed that recall for injuries with minimal disability dropped more than 7 fold over 12 months, whereas recall for injuries with disability lasting 7–29 days decreased 3·6 fold in 12 months, and recall for injuries producing disability for 30 days or more did not decay.[35] The injury numbers for Baghdad may be additionally distorted by a survivor bias where households migrate, are destroyed, or break apart. It is likely households were reluctant to report certain injuries such as self-poisonings or other suicide behaviors, and other injuries with social implications, or feared consequences of reporting events.

The problems with recall for a 11 year study are real. Several off-setting factors influence the effect of recall bias on findings. The proportion of injured people with disability remained about the same over the entire recall period. Significant recall bias would lead to a decreasing proportion of disability reported over time, as people would recall a greater proportion of minor injuries in the more recent past. Hence, injured people from 2013 and 2014 are reporting many disabilities from injuries less than one year ago. With time the transient disabilities from these injuries, especially milder ones, could be lost to recall. Thus, the rise in injury rates in 2013–4 might still be in large part due to the effect of recall bias, rather than actual increases in injury incidence rates. It is noteworthy that there are different trends for non-fatal injuries (both with and without disabilities) for intentional vs. unintentional injuries (as seen in Fig 3). If the rise in unintentional injuries (and especially falls) were due mostly to recall bias, one would expect a similar rise for intentional injuries, which is not seen. This tends to reinforce the validity of the finding of increases in unintentional injury rates in recent years.

Data presented are reported by the head of household and other adults, and are subject to many biases. Unlike mortality surveys which checked death certificates, there were no verification methods for injuries. Neither was there the opportunity to compare these findings to pre-2003 data.

## Conclusions

Intentional violence added substantially to the burden of unintentional violence during the 11 years of conflict in Baghdad. More than a third of intentional injuries ended fatally, and this pattern followed the phases of the conflict. Unintentional injuries may also have been influenced by the conflict through deterioration of infrastructure, and injury outcomes by the state of health services. The high proportion of injuries resulting in disabilities means that the impact of these injuries is likely to be felt for many years. Measuring the consequences of conflict must look beyond just mortality estimates.
